# mRNA Expression of Interferon Regulatory Factors during Acute Rejection of Liver Transplants in Patients with Autoimmune Hepatitis

**Published:** 2018-02-01

**Authors:** M. Nasiri, B. Geramizadeh, S. H. Nabavizadeh, S. A. Male-Hosseini, M. H. Karimi, I. Saadat

**Affiliations:** 1 Department of Biology, College of Sciences, Shiraz University, Shiraz, Iran; 2 Transplant Research Center, Shiraz University of Medical Sciences, Shiraz, Iran; 3 Cellular and Molecular Research Center, Yasuj University of Medical Sciences, Yasuj, Iran

**Keywords:** Interferon regulatory factor, Liver transplantation, Graft rejection, Hepatitis, autoimmune, Toll like receptor, RNA, Messenger, transcription factors

## Abstract

**Background:**

Interferon regulatory factors (IRFs) can play a critical role in the regulation of many facets of innate and adaptive immune responses through transcriptional activation of type I interferons, other proinflammatory cytokines, and chemokines. However, their roles in transplantation immunity still remain to be elucidated.

**Objective:**

To evaluate the time course of mRNA expression of all 9 members of IRFs family of transcription factors during liver allograft acute rejection.

**Methods:**

Blood samples of 19 patients with autoimmune hepatitis receiving liver transplants were collected on days 1, 3, 5, and 7 post-transplantation. The patients were followed for 6 months after transplantation and divided into two groups of acute rejection (AR) (n=4) and non-acute rejection (non-AR) (n=15).

**Results:**

All of the studied transcription factors were down-regulated in AR-group on days 3, 5, and 7 post-transplantation compared to non-AR group. The mean±SEM IRF5 on day 7 post-transplantation was significantly (p=0.005) lower in AR-group than in non-AR group (0.7±0.21 *vs*. 1.91±0.27, respectively); expression of other IRFs family members was not significantly different between the two groups on days 3, 5, and 7 post-transplantation.

**Conclusion:**

IRF5 may have an important role during the acute rejection of liver transplants.

## INTRODUCTION

Autoimmune hepatitis (AIH) is a chronic inflammatory disease with unknown etiology. It has a mean incidence of 1.9 cases per 100,000 people per year and a prevalence of 16.9 cases per 100,000 people [1]. Liver transplantation (LT) is the final therapeutic option for patients with AIH presenting with fulminant hepatic failure [2]. In spite of improved immunosuppressive protocols after LT, the incidence of acute rejection (AR) in AIH patients have been remained in the range of 20% to 88% [3-6].

Whereas T cell responses are both necessary and sufficient for acute allograft rejection, most of researchers have been focused on adaptive immune responses in transplantation immunology [7]. During the last decade, several studies revealed the role of innate immunity as a critical trigger for adaptive immune responses in AR, through recognition and response to endogenous ligands released by damaged or killed cells during tissue injury or disease [7-9]. 

Involvement of the innate immunity in AR is arising from the important role of Toll-like receptors (TLRs) as the first responders to danger signals [10]. In recent years, this is supported by several reports that TLRs, which recognize pathogen-associated molecular patterns (PAMPs) on different micro-organisms, can prevent allograft tolerance by recognizing endogenous ligands released after transplantation and producing proinflammatory cytokines, chemokines, and type I interferon (IFN) [10-12]. 

Signaling through TLRs can be categorized into two pathways: the MyD88 (myeloid differentiation primary-response protein 88) dependent pathway and TRIF (TIR domain containing adaptor inducing IFN-β) dependent pathway [13]. Both of these pathways are leading to the activation of three major downstream molecules: Nuclear factor κB (NF-κB), mitogen-activated protein kinases (MAPKs), and interferon regulatory factors (IRFs) [14]. 

IRFs family of transcription factors consists of nine members in humans: IRF1, IRF2, IRF3, IRF4 (also known as PIP, LSIRF, or ICSAT), IRF5, IRF6, IRF7, IRF8 (also known as ICSBP), and IRF9 (also known as ISGF3γ) [14]. Each IRF contains a N-terminal DNA binding domain (DBD) that can recognize IFN-stimulated response elements (ISRE) located in the promoters of type-I IFN genes, responding genes to type-I IFNs signaling, and genes that are involved in immunity and oncogenesis [15]. The C-terminal region of IRFs, except for IRF1 and IRF2, possesses an IRF-associated domain (IAD) that is responsible for homometric and heteromeric interactions with other family members or other transcription factors [15]. Therefore, IRFs can play a critical role in the regulation of many facets of innate and adaptive immune responses downstreaming TLRs signaling through transcriptional activation of type-I IFNs, other proinflammatory cytokines, and chemokines [15, 16]. 

Several gene expression profiling studies have so far reported the role of IRFs (such as IRF1, IRF3, IRF5, IRF8, and IRF9) in acute allograft rejection [17-20]. Also, a few gene array analyses of rat models of liver transplantation tolerance, indicated the role of IRF1 during tolerance induction [21, 22]. Recently, Yu, *et al*, demonstrated that single nucleotide polymorphism in promoter region of IRF5 encoding gene, is correlated with acute rejection of liver allograft [23]. In addition, high-throughput genetic studies of primary biliary cirrhosis (PBC) associated IRF5 and IRF8 with the pathogenicity of autoimmune liver diseases [6, 24-26]. Nevertheless, there is no evidence for the involvement of IRFs in acute rejection after liver transplantation in AIH patients. 

This prospective study tried to clarify the role of IRFs family of transcription factors, in acute rejection after liver transplantation in AIH patients. We evaluated the time course of mRNA expression levels of IRFs in peripheral blood mononuclear cells (PBMCs) of patients with AIH who had acute and non-acute rejection of liver transplants.

## MATERIAL AND METHODS

From May 2012 to March 2014, 20 Iranian adult female patients, who satisfied the international criteria for AIH [27], and received orthotopic liver transplantation (OLT), at the Transplantation Center of Namazi Hospital affiliated to Shiraz University of Medical Sciences, Shiraz, Iran, were selected for this study. One patient expired during the first week of transplantation, and was thus excluded from the study. Blood samples from each patient were collected, using EDTA containing tubes on day 1, 3, 5, and 7 post-transplantation. All patients were followed for six months of OLT and divided into two groups according to their hepatic biopsy result: group I (AR-group) composing of four patients with at least one AR episode during the six months, and group II (non-AR group) composing of 15 patients without any AR episodes during the study period. The diagnoses of AR was based on well-accepted criteria including increased serum transaminases and total bilirubin levels in the absence of vascular problems or biliary obstruction. The diagnosis was confirmed by histological findings after liver biopsy, according to the criteria set for AR described by the Banff schema [28]. 

The study protocol was approved by the Ethics Committee of both Shiraz University and Shiraz University of Medical Sciences, based on the study protocol conformed to the ethical guidelines of the 1975 declaration of Helsinki. 

Immunosuppressant Regimen

All recipients received the routine immunosuppression regimen consisted of tacrolimus or cyclosporine (CsA) with mycophenolate mofetil and steroids. Briefly, the drug dosage was adjusted to maintain target therapeutic blood levels of 200 ng/mL for CsA (5 mg/kg/day), or 10 ng/mL for tacrolimus. The patients with AR episodes received high dose (500 mg) methylprednisolone for three consecutive days.

RNA Extraction and cDNA Synthesis

Total RNA was isolated from 1.5 mL fresh whole blood of recipients, immediately after sample collection, using QIAamp RNA blood mini kit (Qiagen; Hilden, Germany) according to the manufacturer’s instructions. The purity and integrity of RNA was evaluated through measuring the optical density 260/280 ratio by spectrophotometric analysis. Then 500 ng of RNA samples with ratio above the 1.8 were used for cDNA synthesis by PrimeScript 1^st^ strand cDNA Synthesis Kit (Takara, Japan), according to manufacturer’s protocol.

Quantitative Real-time PCR

The mRNA expression levels of IRFs were determined by StepOnePlus real-time PCR instrument (Applied Biosystems, USA), using SYBR Premix Ex Taq II kit (Takara, Japan). The gene-specific primer sets were designed to span introns or cross exon/exon junctions, using AlleleID software *ver* 7.8 (Premier Biosoft Int, CA, USA). Genomic DNA amplification was not appeared in our qPCR reactions, containing not reverse transcribed RNA as template. Real-time PCR primer sequences and conditions are presented in Table 1, according to the MIQE guideline [29]. RPL13a gene was used as the internal control. Two-step real-time PCR was performed in 10 µL total volume of reaction, including 5 µL of SYBR Premix, 0.4 µL of each forward and reverse primer (final concentration of 0.4 µM), 0.2 µL of ROX dye, and 4 µL of diluted cDNA as a template (final concentration of 10 ng/reaction), for 45 cycles with initial denaturation/activation for 30 s at 95 °C, 5 s denaturation at 95 °C, and 45 s annealing/extension at 60 °C. Expression fold changes were calculated relative to day 1, using ΔΔCT method. The specificity of each amplification reaction was confirmed by a melting-curve analysis. In order to monitor for “primer-dimer” formation, a no-template control (NTC) tube for each gene was included in all experiments.

**Table 1:** Details of primers condition and amplicons

Gene symbol	Accession number	Forward and revers primer (5’ to 3’)	Amplicon size (bp)	Intron spanning	Crossing exon/exon junction
*IRF1*	NM_002198	CTCCACCTCTGAAGCTACAA	133	Yes	Yes
		TCCAGGTTCATTGAGTAGGT			
*IRF2*	NM_002199	GGCTCAAGTGGCTTAACAA	135	Yes	Yes
		CTGGTTGATGCTTTCCTGTAT			
*IRF3* *	NM_001571 NM_001197128 NM_001197127 NM_001197125 NM_001197126 NM_001197124 NM_001197123 NM_001197122	TCGTGATGGTCAAGGTTGT	94	No	Yes
		AGGTCCACAGTATTCTCCAG			
*IRF4* *	NM_002460 NM_001195286	AGCAGTTCTTGTCAGAGC	135	No	Yes
		GTTCTACGTGAGCTGTGATG			
*IRF5* *	NM_001098627 NM_001098630 NM_001098629 NM_032643	ATGCTGCCTCTGACCGA	141	No	Yes
		GCCGAAGAGTTCCACCTG			
*IRF6* *	NM_006147 NM_001206696	CTCATCTTGGTTCAGGTCATTC	95	No	Yes
		CGGACACTGCCACTATCA			
*IRF7* *	NM_001572 NM_004031 NM_004029	GCAAGTGCAAGGTGTACTG	131	No	Yes
		CACCAGCTCTTGGAAGAAGA			
*IRF8*	NM_002163	AGCCTTCTGTGGACGATTAC	167	Yes	Yes
		CTGGGAGAATGCTGAATGGT			
*IRF9*	NM_006084	TGAGCCACAGGAAGTTACA	103	Yes	yes
		GAGCAGCAGTGAGTAGTCT			
*RPL13a*	NM_012423	GATAAGAAACCCTGCGACAAA	193	Yes	No
		AGAAATTGCCAGAAATGTTGATG			

IRF: Interferon regulatory factor; RPL13a: Ribosomal protein L13a

* Several transcript variants were amplified using these primer pairs.

Statistical Analysis

The statistical significance of differences in the measured gene expression levels between AR and non-AR groups was evaluated by Mann-Whitney U test, using SPSS *ver* 16 (SPSS Inc, Chicago, IL, USA). Data are presented as mean±standard error of the mean (SEM). A p value <0.05 was considered statistically significant.

## RESULTS

Among 19 patients enrolled in this study, four with a mean age of 35 (range: 24–51) years experienced one AR episode during the six months of OLT; 15 patients with a mean age of 34.8 (range: 14–46) years did not experience AR during the study period. All AR episodes occurred within the first month post-transplantation. There were no significant difference in age, BMI, MELD score, total cold ischemic time, warm ischemic time, donor age, and sex, between AR and non-AR groups (Table 2). No significant difference was also observed in mean serum tacrolimus levels measured on days 3 and 7 post-transplantation, between AR and non-AR recipients (Table 2).

**Table 2:** Characteristics of patients, all with autoimmune hepatitis. Values are mean±SEM.

	Acute rejection (n=4)	Non-acute rejection (n=15)	p value
Age (yrs)	35±5.7	34.8±3.63	0.92
BMI (kg/m^2^)	20.7±2.3	25.39±1.08	0.067
MELD score	24.25±4.32	23.25±1.21	0.76
Donor age	30.25±9.92	31.13±2.8	0.48
Donor sex (M/F)	3/1	13/2	0.58
Total cold ischemic time (min)	525±28.72	450±48.51	0.52
Warm ischemic time (min)	31.25±2.39	36.07±2.17	0.28
Serum tacrolimus level (ng/mL)*			
Day 3 post-transplantation	5.37±0.67	6.21±0.4	0.50
Day 7 post-transplantation	8.73±1.83	8.47±0.74	0.71

BMI: Body mass index; AIH: Autoimmune hepatitis.

* Data for serum tacrolimus level of patients on days 1 and 5 post-transplantation were not sufficient for analysis.

Expression of IRFs in AR *vs*. Non-AR group

**Figure 1:**
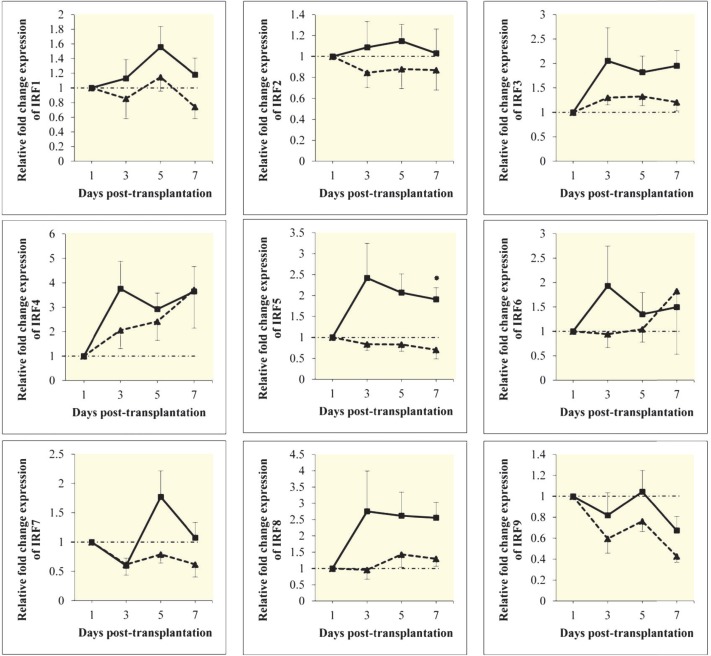
Mean mRNA expression levels of all nine members of IRFs family during one week post-transplantation in liver graft recipients who developed acute rejection (dashed line) and those who did not (solid line). Error bars represent the standard error of the mean. An asterisk indicates a significant difference (p=0.005) between the two groups

Blood mRNA levels of IRF3, IRF4, IRF6 and IRF8 were upregulated on days 3, 5, and 7 post-transplantation relative to day 1 in both AR and non-AR groups. The mean expression levels of these transcription factors in AR group were however reduced compared to non-AR group. We found that the mRNA expression levels for IRF1, IRF2, IRF5, and IRF7, were almost downregulated in AR group and upregulated in non-AR group, on days 3, 5, and 7 relative to day 1. IRF9 had almost downregulation in both groups. Mean expression levels of IRF1, IRF2, IRF5, IRF7, and IRF9, decreased in AR group compared to non-AR group. The mean±SEM IRF5 was significantly (p=0.005) lower on day 7 post-transplantation in AR-group than in non-AR group (0.7±0.21 *vs.* 1.91±0.27, respectively). Expression levels of other IRFs family members were not significantly different between the two groups on days 3, 5, and 7 post-transplantation (Fig 1).

## DISCUSSION

In our study, all other underlying liver diseases such as viral infections, metabolic diseases, non-alcoholic fatty liver diseases (NAFLD), as well as other types of liver autoimmune diseases were excluded. Therefore, it might be assumed that the comparison of IRFs expression levels between AR and non-AR groups, with the same underlying disease, represented the functional impact of these transcription factors in AR after OLT.

Our results demonstrated that the mRNA expression of all nine members of this family decreased in AR compared to non-AR group on days 3, 5, and 7 post-transplantation, but only the down-regulation of IRF5 on day 7 post-transplantation was significant. 

It is well established that danger signals, released from donor organ as a result of ischemia-reperfusion injury, tissue damages, and hepatic phase of the transplant procedure, can activate Toll-like receptors, especially TLR4 [7, 10, 12, 30-32]. Activation of TLR4 leads to the activation and maturation of dendritic cells with subsequent secretion of various cytokines and chemokines and initiation of an adaptive immune response during the pre-transplantation period [33].

Testro, *et al* [34], reported that the expression levels of TLR4 upon PBMCs at pre-transplantation time were significantly upregulated in those who rejected their liver grafts compared to those who did not, but it was significantly down-regulated on day 7 post-transplantation in patients with rejection due to activation of negative regulatory response after an initial burst of TLR4-mediated signaling [35, 36]. Several IRF family members, especially IRF1, IRF3, IRF5, IRF7, and IRF8 can activate downstreaming of TLR4 to induce inflammatory responses [15]. A previous study by Takaoka, *et al*, showed that activation of TLR4 invokes nuclear translocation of IRF5 [37]. Unlike IRF-3 and IRF-7, IRF-5 is generally involved in downstreaming of TLR4-MyD88 signaling pathway, as a master transcription factor in the transcriptional activation of inflammatory cytokine genes [37]. Although little direct evidence has shown the involvement of IRF5 in AR, its capability of transcriptionally activating pro-inflammatory genes through TLR4 cascade implies potential roles. So significant post-transplantation downregulation of IRF5 (not other IRF family members) on day 7, in patients who developed AR, is well conformed to the downregulation pattern of TLR4 on day 7 post-transplantation and suggests a potential role for IRF5 downstreaming of TLR4 signaling in AR of liver transplants. 

In conclusion, despite of our small study sample size and limitation in pre-transplantation sample collection, this study, for the first time, tried to represent evidence for the role of IRF5 in liver transplant AR. Further studies are needed to elucidate the functional impact of this transcription factor on liver transplant AR.
